# FAK-Mediated Signaling Controls Amyloid Beta Overload, Learning and Memory Deficits in a Mouse Model of Alzheimer’s Disease

**DOI:** 10.3390/ijms23169055

**Published:** 2022-08-13

**Authors:** Bisan Saleh, Kolluru D. Srikanth, Tal Sneh, Lambert Yue, Steven Pelech, Evan Elliott, Hava Gil-Henn

**Affiliations:** 1 Cytoskeletal Signaling Laboratory, The Azrieli Faculty of Medicine, Bar-Ilan University, Safed 1311502, Israel; 2 Department of Medicine, Division of Neurology, University of British Columbia, Vancouver, BC V6T 2B5, Canada; 3 Kinexus Bioinformatics Corporation, Suite 1, 8755 Ash Street, Vancouver, BC V6P 6T3, Canada; 4 Molecular and Behavioral Neurosciences Laboratory, The Azrieli Faculty of Medicine, Bar-Ilan University, Safed 1311502, Israel

**Keywords:** Alzheimer’s disease, FAK, amyloid plaques, learning and memory, proteomics, signaling networks

## Abstract

The non-receptor focal adhesion kinase (FAK) is highly expressed in the central nervous system during development, where it regulates neurite outgrowth and axon guidance, but its role in the adult healthy and diseased brain, specifically in Alzheimer’s disease (AD), is largely unknown. Using the 3xTg-AD mouse model, which carries three mutations associated with familial Alzheimer’s disease (APP KM670/671NL Swedish, PSEN1 M146V, MAPT P301L) and develops age-related progressive neuropathology including amyloid plaques and Tau tangles, we describe here, for the first time, the in vivo role of FAK in AD pathology. Our data demonstrate that while site-specific knockdown in the hippocampi of 3xTg-AD mice has no effect on learning and memory, hippocampal overexpression of the protein leads to a significant decrease in learning and memory capabilities, which is accompanied by a significant increase in amyloid β (Aβ) load. Furthermore, neuronal morphology is altered following hippocampal overexpression of FAK in these mice. High-throughput proteomics analysis of total and phosphorylated proteins in the hippocampi of FAK overexpressing mice indicates that FAK controls AD-like phenotypes by inhibiting cytoskeletal remodeling in neurons which results in morphological changes, by increasing Tau hyperphosphorylation, and by blocking astrocyte differentiation. FAK activates cell cycle re-entry and consequent cell death while downregulating insulin signaling, thereby increasing insulin resistance and leading to oxidative stress. Our data provide an overview of the signaling networks by which FAK regulates AD pathology and identify FAK as a novel therapeutic target for treating AD.

## 1. Introduction 

Alzheimer’s disease (AD) is a progressive neurodegenerative disorder, characterized by impaired memory, apathy, depression, and specific neuropathological changes that include the accumulation of amyloid plaques and neurofibrillary tangles. The disorder mainly affects older people with a gradual onset and comprises between 50% and 75% of all dementia cases as the 6th leading cause of death, affecting more than 5.7 million people at all ages in the USA and more than 47 million people worldwide [1,2]. Advances in medicine that lead to longer life expectancy, along with the long duration of illness before death from AD contribute significantly to the social and economic burden of the disease [3]. Despite considerable research to understand the pathogenesis and causative mechanisms that underlie AD, many questions still need to be addressed. Understanding the molecular basis of AD can assist in the development of targeted treatment of the disease, that currently has no cure [4]. 

The non-receptor focal adhesion kinase (FAK; gene name *PTK2*) is highly expressed in the central nervous system during development and in the adult brain [5], particularly enriched in the cortex and hippocampus, brain regions with pivotal importance for learning and memory. Within the brain, FAK is localized in axons and dendrites of neurons and in intermediate filaments of astrocytes [6], is regulated by neurotransmitters, and is implicated in synaptic plasticity and ion channel regulation [7]. 

Knockdown of FAK in hippocampal neurons shows a significant decrease in mature mushroom-like dendritic spines and an increase in immature filopodia dendritic spines, indicating that FAK may have a role in the formation or stabilization of mature spines [8,9]. Conditional knockout of FAK in the brain results in cortical abnormalities that are associated with dystroglycanopathies [10]. Moreover, the FAK inhibitor Y15 controls neuronal growth, and is involved in the regulation of synaptic plasticity and hippocampus-dependent spatial learning and memory in vivo [11]. Considered together, these data highlight a critical role of FAK in the functioning of neuronal systems. However, the specific contribution of FAK to molecular pathways underlying neuronal growth, synaptic function and plasticity, and consequent mouse behavior and its specific functions in normal and diseased brains remain poorly understood.

Altered phosphorylation of FAK and the protein tyrosine kinase Src is observed in human brains of AD patients [12], and alterations in tyrosine phosphorylation in brains of patients with the disease lead to increased FAK and Tau (MAPT) phosphorylation in response to amyloid β (Aβ) exposure [13]. Moreover, it was previously demonstrated that Aβ oligomer treatment of primary human and rat cortical neuronal cultures leads to association of the protein-tyrosine kinase Fyn with FAK and consequent increase in FAK tyrosine phosphorylation and activation [13], which is involved in amyloid precursor protein (APP)-mediated neuronal injury [14]. 

FAK is known to have a crucial role in regulating insulin signaling and insulin resistance in peripheral tissues. In contrast, in neuronal cells FAK acts as a negative regulator of insulin signaling and altered phosphorylation of FAK is observed in neuronal insulin resistance [15]. Along these lines, FAK has been detected in dystrophic neurons that surround amyloid deposits in AD brains, which may indicate that FAK activity is involved in Aβ-induced neuronal cell death [16]. Despite these and other data implicating a role for FAK in AD, the specific mechanisms and pathways by which FAK regulates AD pathology are largely unknown. 

Using the 3xTg-AD mouse model, we show here, for the first time, that hippocampal overexpression of FAK, but not its deletion, has a deleterious effect on learning and memory. Moreover, we show that overexpression of FAK leads to a significant increase in Aβ load, which is accompanied by reduction in astrogliosis and alterations in hippocampal neuron morphology. Using high-throughput total- and phospho-proteomics combined with bioinformatics analysis, we characterize the molecular and signaling cascades that may impact AD progression, and which include cytoskeletal signaling, insulin signaling and resistance, cell cycle re-entry, and consequent neuronal cell death. Based on this analysis, we suggest a model that explains the behavioral, neuronal, and astrocyte-related phenotypes observed in this study.

## 2. Results

### 2.1. Hippocampal Depletion of FAK in 3xTg-AD Mice Does Not Significantly Affect Their Learning and Memory Capabilities

**Figure 1:**
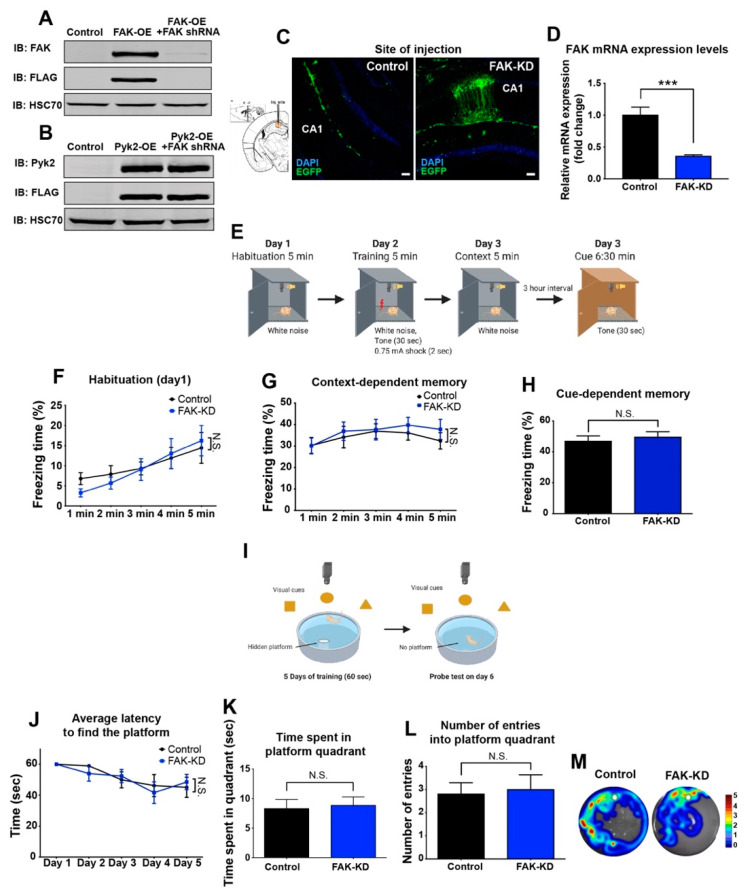
Hippocampal depletion of FAK in 3xTg-AD mice does not significantly affect their learning and memory capabilities. (**A**) HEK293T cells were transfected with FLAG-tagged control viral vector, vector expressing FAK (FAK-OE) or vector expressing FAK and a knockdown vector (FAK-KD). Expression and knockdown were verified by immunoblot analysis. (**B**) HEK293T cells were transfected with FLAG-tagged control viral vector, vector expressing Pyk2 (Pyk2-OE) or vector expressing a knockdown vector for FAK (FAK-KD). The specificity of FAK-KD vector to FAK only and not to Pyk2 was verified by immunoblot analysis. (**C**) Left, diagram of the coordinates for site injection of CA1 region from the Allen mouse brain atlas. Right, representative confocal images of control and FAK-KD hippocampi. Green: Enhanced green fluorescent protein (EGFP); blue: 4 w, 6-diamidino-2-phenylindole (DAPI) DNA stain. Scale bar, 100 μm. (**D**) FAK mRNA expression levels in hippocampi of 3xTg-AD mice injected with control or FAK shRNA lentiviral vector. *N* = 5 mice per group, *** *p* = 0.0009 by an unpaired Student’s *t*-test. (**E**) Schematic diagram of the fear conditioning paradigm. (**F**) Freezing response during habituation on the first day of fear conditioning paradigm. *p* = N.S. by two-way ANOVA with the Bonferroni post hoc test. (**G**) Freezing response in context-dependent memory test. *p* = N.S. by two-way ANOVA with the Bonferroni post hoc test. (**H**) Freezing response in cue-dependent memory test. *p* = N.S. by an unpaired Student’s *t*-test. (**I**) Schematic diagram of the Morris water maze paradigm. (**J**) Average latency to find the platform during 5 days of acquisition phase. *p* = N.S. by two-way ANOVA with the Bonferroni post hoc test. (**K**) Time spent in the platform quadrant during the probe test day. *p* = N.S. by an unpaired Student’s *t*-test. (**L**) Analysis of the number of entries into the platform quadrant during the probe test day. *p* = N.S. by an unpaired Student’s *t*-test. (**M**) Representative heatmaps of control and FAK-KD mice during the probe test day. Location of the platform is indicated by the white circle.

To explore the role of FAK in AD, we used the triple transgenic mouse model 3xTg-AD. These mice express PS1_M146V_, APP_SWE_, and Tau_P301L_ and develop both plaque and tangle pathologies in their brain, thereby exhibiting deficits in synaptic plasticity. As a result, these mice provide a valuable model for studying AD pathology. To examine the effect of depletion of FAK on learning and memory in the 3xTg-AD mice, a lentivirus containing shRNA sequence specific for the *Ptk2* gene (FAK-KD) or a control virus was introduced into the hippocampi of 8-month-old 3xTg-AD mice by stereotaxic injection (Figure 1A–D). Following a recovery period of 3 weeks, we used two classical paradigms. 

The fear conditioning paradigm uses an aversive stimulus (foot electric shock) with context and tone stimuli and tests the context- and cue-dependent fear memory, respectively (Figure 1E). In the contextual fear conditioning paradigm, FAK-KD mice showed no difference in freezing levels on the first day of the test, and no baseline changes were observed between FAK-KD and control mice (habituation; Figure 1F). When mice were re-introduced to the context that they have previously experienced, they showed no differences in freezing levels compared with control virus-injected mice (Figure 1G). In the tone-dependent cued fear conditioning paradigm, FAK-KD mice showed no differences in freezing levels when presented with tones 1 d after pairing of the tone to the shock (Figure 1H). 

The Morris water maze paradigm measures the spatial memory of the mouse (Figure 1I). In this assay, FAK-KD mice demonstrated no differences in latency to find the hidden platform during the 5 d acquisition phase, compared with control mice (Figure 1J). During the probe trial, in which the hidden platform is removed from the tank (day 6), FAK-KD mice showed no significant differences in the time spent on the platform as well as in the number of visits into the platform quadrant (Figure 1K–M). Collectively, these results demonstrate that hippocampal knockdown of FAK in 3xTg-AD mice does not affect their learning and memory capabilities.

### 2.2. Overexpression of FAK in 3xTg-AD Mouse Hippocampus Leads to Significant Impairment in Learning and Memory

**Figure 2:**
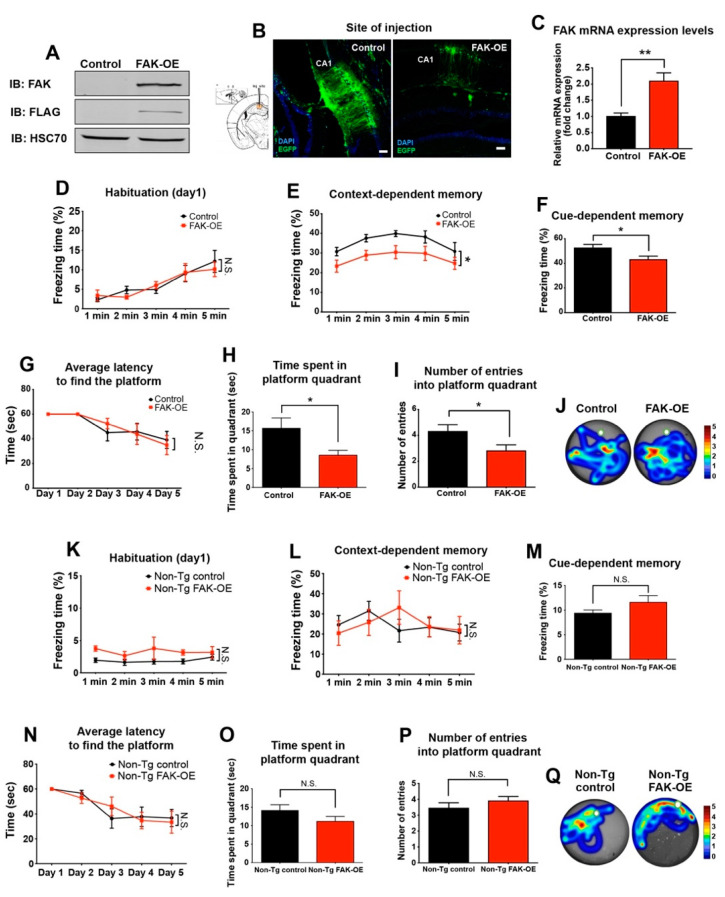
Overexpression of FAK in 3xTg-AD mouse hippocampus leads to significant impairment in learning and memory. Panels (**A**–**J**), 3XTg-AD mice; panels (**K**–**P**), non-Tg, wild-type mice. (**A**) HEK293T cells were transfected with FLAG-tagged control viral vector or a vector expressing FAK (FAK-OE), expression was verified by immunoblot analysis. (**B**) Left, diagram of the coordinates for site injection of CA1 region from the Allen mouse brain atlas. Right, representative confocal images of control and FAK-OE hippocampi. Green, EGFP; blue, DAPI. Scale bar, 100 μm. **(C)** FAK mRNA expression levels in hippocampi of 3xTg-AD mice injected with control or FAK expressing lentiviral vector. *N* = 5 mice per group, ** *p* = 0.0043 by an unpaired Student’s *t*-test. (**D**) Freezing response during habituation on the first day of fear conditioning paradigm. *p* = N.S. by two-way ANOVA with Bonferroni post hoc test. (**E**) Freezing response in context-dependent memory test. * *p* = 0.0175 by two-way ANOVA with the Bonferroni post hoc test. (**F**) Freezing response in cue-dependent memory test. * *p* = 0.0364 by an unpaired Student’s *t*-test. (**G**) Average latency to find the platform during 5 d of acquisition phase. *p* = N.S. by two-way ANOVA with the Bonferroni post hoc test. (**H**) Time spent in the platform quadrant during the probe test day. * *p* = 0.0281 by an unpaired Student’s *t*-test. (**I**) Analysis of the number of entries into the platform quadrant during the probe test day. * *p* = 0.0452 by an unpaired Student’s *t*-test. (**J**) Representative heatmaps of control and FAK-OE mice during the probe test day. *n* = 10 in each control and FAK-OE groups. (**K**) Freezing response during habituation on the first day of fear conditioning paradigm. *p* = N.S. by two-way ANOVA with the Bonferroni post hoc test. (**L**) Freezing response in context-dependent memory test. *p* = N.S. by two-way ANOVA with the Bonferroni post hoc test. (**M**) Freezing response in cue-dependent memory test. *p* = N.S. by an unpaired Student’s *t*-test. (**N**) Average latency to find the platform during 5 d of acquisition phase. *p* = N.S. by two-way ANOVA with the Bonferroni post hoc test. (**O**) Time spent in the platform quadrant during the probe test day. *p* = N.S. by an unpaired Student’s *t*-test. (**P**) Analysis of the number of entries into the platform quadrant during the probe test day. *p* = N.S. by an unpaired Student’s *t*-test. (**Q**) Representative heatmaps of control and FAK-OE mice during the probe test day. *N* = 10 in each control and FAK-OE groups.

To further determine whether overexpression of FAK has any effect on learning and memory in 3xTg-AD mice, the fear conditioning paradigm was performed on mice that were stereotaxically injected into their hippocampus with a lentiviral vector expressing the *Ptk2* gene (FAK-OE) or a control virus (Figure 2A–C). To examine whether FAK-OE mice have baseline differences in their memory compared with control mice, they were introduced into a new chamber at the first day of the test and their freezing activity was measured. While FAK-OE mice showed no differences in their freezing activity during the first day of the paradigm (Figure 2D), they displayed significantly lower freezing levels compared with control mice on the following days of the paradigm, in both context- and cue-dependent memory (Figure 2E,F).

Further examination of control and FAK-OE mice in the Morris water maze paradigm demonstrated no significant differences in the latency to find the platform (Figure 2G). However, a significant decrease in the time spent in the platform quadrant and in the number of entries into the platform quadrant was observed (Figure 2H–J). Considered together, these observations indicate that overexpression of FAK in the hippocampi of 3xTg-AD mice leads to severe impairments in their memory and learning capabilities.

### 2.3. FAK Overexpression in Hippocampi of Non-Transgenic Aged Mice Does Not Affect Their Learning and Memory Capabilities

To examine the effect of FAK overexpression in normal, non-transgenic (non-Tg) aged mice, FAK overexpressing lentiviral vector was injected into the hippocampi of 8-month-old non-transgenic (non-Tg) wild-type C57BL/6 mice. Following a recovery period of 3 weeks, mice were subjected to the fear conditioning and Morris water maze paradigms. Non-Tg FAK-OE mice showed no significant differences in freezing levels in the first day of habituation, and in context- and cue-dependent memory compared with control lentiviral vector injected mice (Figure 1K–M). In addition, non-Tg FAK-OE mice showed no significant differences in the average latency to find the hidden platform, the time spent in the platform quadrant or in the number of entries into the platform quadrant (Figure 1N–Q). These data support the notion that FAK overexpression does not affect learning and memory behaviors that may be changed due to aging in a wild-type, non-pathological condition.

### 2.4. Hippocampal Overexpression of FAK Significantly Increases Amyloid Plaque Accumulation

**Figure 3:**
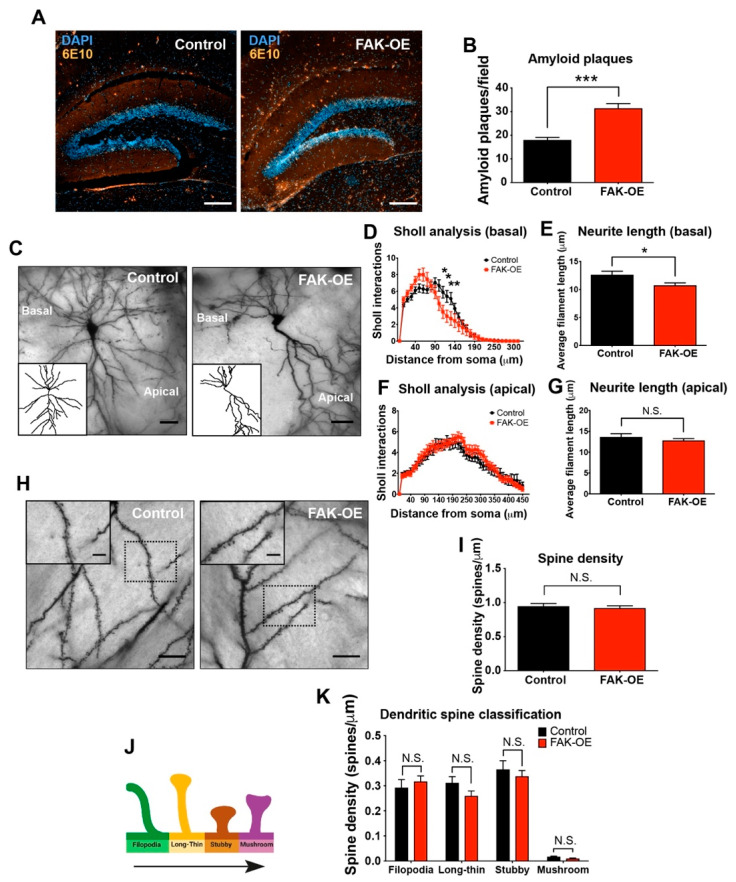
Hippocampal FAK overexpression increases amyloid plaque accumulation and reduces neuronal basal dendritic arborization. (**A**) Representative images of control and FAK-OE hippocampi labeled for amyloid plaques (orange, 6E10) and for cell nuclei (blue, DAPI). Scale bar, 200 μm. (**B**) Quantification of amyloid plaque number in control and FAK-OE hippocampi. *** *p* < 0.001 by an unpaired Student’s *t*-test. *N* = 10 fields per group. (**C**) Representative images of CA1 hippocampus pyramidal neurons from control and FAK-OE mice. (**D**) Sholl interaction analysis of basal dendritic intersections of control and FAK-OE pyramidal neurons. * *p* < 0.05, ** *p* < 0.01 by two-way ANOVA with the Bonferroni post hoc test. (**E**) Average dendritic filament length of basal regions of control and FAK-OE pyramidal neurons. *p* = 0.0374 by an unpaired Student’s *t*-test. (**F**) Sholl interaction analysis of apical dendritic intersections of control and FAK-OE pyramidal neurons. *p* = N.S. by two-way ANOVA with the Bonferroni post hoc test. (**G**) Average dendritic filament length of apical regions of control and FAK-OE pyramidal neurons. *p* = N.S. by an unpaired Student’s *t*-test. *N* = 45 neurons from five mice in each group. (**H**) Representative images of dendrites with spines of hippocampal pyramidal neurons of control and FAK-OE mice. Scale bar, 5 μm. (**I**) Quantification of number of spines (spine density) in arbors from control and FAK-OE pyramidal neurons. *p* = N.S. by an unpaired Student’s *t*-test. (**K**) Quantification of spine types (filopodia, long thin, stubby, and mushroom); (**J**) in control and FAK-OE neurons. *p* = N.S. by an unpaired Student’s *t*-test between control and FAK-OE in each type. *N* = 30 neurons from five mice in each group.

Amyloid plaque accumulation is a hallmark pathological feature of AD. Increased amyloid deposition in the brain has been linked to over-production of APP and Aβ in early onset familial AD cases which are caused by mutations in APP, PSEN1, and PSEN2, a phenotype that has been partially recapitulated in AD-like transgenic mice expressing mutant forms of APP and Aβ [17,18,19,20]. To evaluate the impact of overexpression of FAK on amyloid burden, coronal brain sections of control and FAK-OE 3xTg-AD mice were immunofluorescently labeled for Aβ. FAK overexpression resulted in a significant increase in the number of amyloid plaques within the hippocampi of 3xTg-AD mice, indicating that FAK may be involved in the process of plaque formation and accumulation in the brains of these mice (Figure 3A,B).

### 2.5. FAK Overexpression Shows Reduced Arborization in Basal Regions of Hippocampus Neurons

The brains of affected individuals in AD exhibit significant synapse loss and dendritic arbor regression, and the degree of cognitive and memory impairments in AD patients correlate with the extent of synapse and dendritic arbor loss [21]. Furthermore, FAK is known to regulate neurite outgrowth and branching of developing neurons in healthy brains [9]. To evaluate the impact of overexpression of FAK on the morphology of AD mouse brain neurons, we performed Golgi-Cox staining of hippocampus sections from control and FAK-OE 3xTg-AD mice (Figure 3C). Specifically, we analyzed the pyramidal cells, which are known to play a key role in information processing within cortical and hippocampal circuits and receive multiple inputs into basal and apical dendritic trees [22]. Interestingly, overexpression of FAK in 3xTg-AD mice showed a significant decrease in Sholl analysis interactions as well as in average filament length in basal regions of FAK-OE neurons (Figure 3D,E), but no significant differences were observed in the apical regions of FAK-OE pyramidal neurons (Figure 3F,G). Moreover, no differences were observed in spine densities nor in spine types (filopodia, long-thin, stubby, mushroom) in FAK-OE neurons compared with control neurons (Figure 3H–K). Considered together, these observations indicate that overexpression of FAK in the hippocampi of 3xTg-AD mice contributes to morphological changes in the basal, but not in the apical regions of their pyramidal neurons.

### 2.6. Hippocampal FAK Overexpression Significantly Reduces Astrogliosis

**Figure 4:**
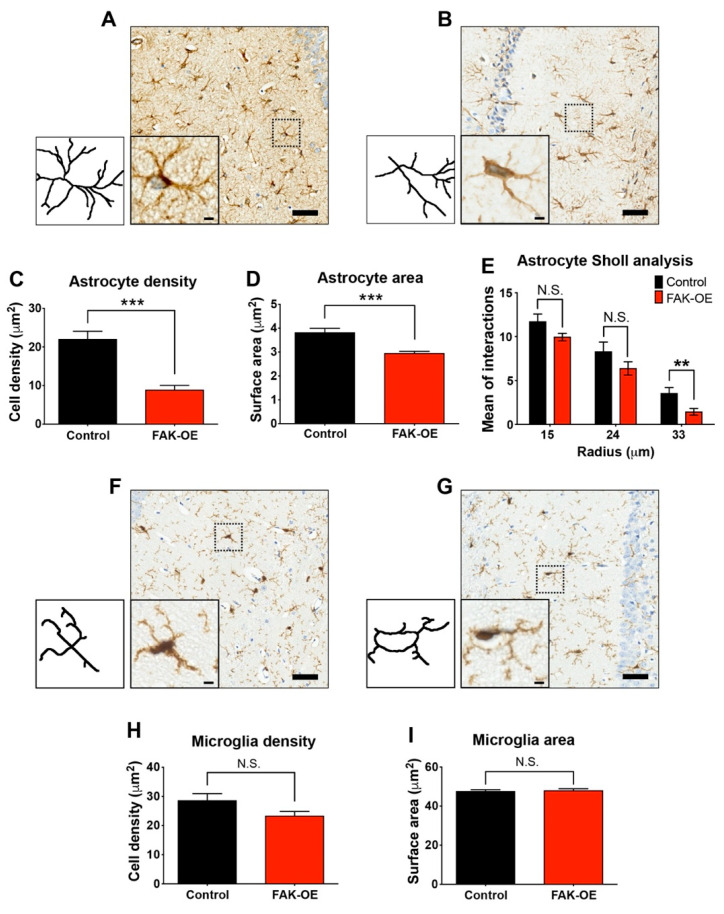
Hippocampal FAK overexpression significantly reduces astrogliosis, but not microgliosis. (**A**,**B**) Representative 20× brightfield images of hippocampi CA1 regions from control and FAK-OE mice labeled with anti-GFAP for astrocytes. Insets: Representative astrocytes, left: Respective trace drawings of representative astrocytes shown in insets. Scale bar, 50 μm (insets: 5 μm). (**C**) Quantification of astrocyte cell density in hippocampi of control and FAK-OE mice. *** *p* < 0.001 by unpaired Student’s *t*-test. (**D**) Quantification of astrocyte surface area *** *p* < 0.001 by unpaired Student’s *t*-test. (**E**) Quantification of Sholl interactions between astrocyte processes in 15, 24, and 33 μm radius from cell center. *p* = N.S., ** *p* = 0.0092 by an unpaired Student’s *t*-test. (**F**,**G**) Representative 2020× brightfield images of hippocampi CA1 regions from control and FAK-OE mice labeled with anti-Iba1 for microglia. Insets: Representative microglia, left: Respective trace drawings of representative microglia shown in insets. Scale bar, 50 μm (insets: 5 μm). (**H**) Quantification of microglia cell density in hippocampi of control and FAK-OE mice. *p* = N.S. by an unpaired Student’s *t*-test. (**I**) Quantification of microglia surface area. *p* = N.S. by an unpaired Student’s *t*-test. *N* = 40 fields from three mice in each group.

Inflammatory reaction accompanies all chronic neurodegenerative processes and is a prominent pathological hallmark of several neurodegenerative disorders including AD. The main contributors of neuroinflammation in AD pathogenesis are astrocytes and microglia, which promote the neuroinflammatory condition independently or in combination with amyloid plaques [23]. In our study, we used lentiviral vectors which mainly infect neurons, but can also infect glial cells [24]. To test the contribution of infected glial cells to neuroinflammation-induced AD-like phenotype of the FAK-OE 3xTg-AD mice, we examined and compared hippocampal astrocytes and microglia by immunohistochemical staining. Histological staining of astrocytes using GFAP as a marker revealed that hippocampal overexpression of FAK leads to reduced astrocyte cell densities, decreased surface area, and a significant decrease in intersections at the 33 μm point of cell radius as measured by Sholl analysis (Figure 4A–E). To examine whether overexpression of FAK within the hippocampus of 3xTg-AD mice affects their enhanced AD-like phenotype by influencing hippocampal microglia, we performed histochemical staining of brain sections from control and FAK-OE mice using Iba1 as a marker. Interestingly, while significantly influencing astrocytes, FAK overexpression did not affect hippocampal microglia cell density or their surface area (Figure 4F–I). Considered together, these data indicate that hippocampal FAK overexpression contributes to the enhanced AD-like phenotype observed in 3xTg-AD mice by affecting both neurons and astrocytes, but not microglial cells.

### 2.7. An in-Lysate Kinase Assay Reveals Novel Targets of FAK in 3xTg-AD Mouse Hippocampus

**Figure 5:**
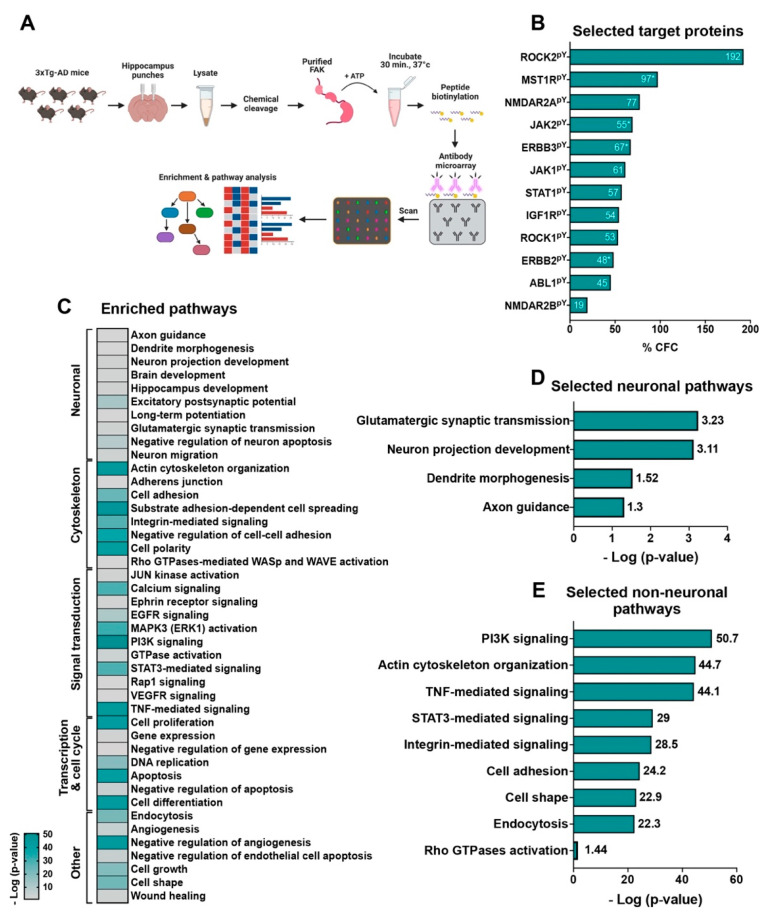
The in-lysate kinase assay reveals novel hippocampal targets of FAK. (**A**) Diagram of the in-lysate FAK kinase assay. (**B**) % CFC values of selected targets of FAK discussed in the text. For proteins with more than one tyrosine phosphorylated, the average % CFC was calculated, and is marked with *. (**C**) Heatmap of enriched pathways from the in-lysate kinase assay. Pathways containing three proteins or more were included in the map. (**D**) Selected neuronal enriched pathways. (**E**) Selected non-neuronal enriched pathways. Pathways with a score of *p* ≤ 0.5 (log *p*-value ≥ 1.3) were selected and are discussed in the text.

To evaluate the mechanistic role of FAK in 3xTg-AD mouse hippocampus, we performed an in-lysate kinase assay. This method measures alterations in tyrosine phosphorylation levels of protein following incubation of hippocampal lysates of 3xTg-AD mice with purified FAK and in the presence of ATP (Figure 5A). In this assay, the chemical cleavage of the hippocampal proteins abolishes the catalytic activities of kinases, phosphatases, and proteases, in order that following the addition of an exogenous active protein kinase may be possible to identify substrates based on their enhanced phosphorylation state, which is revealed by increased binding to phosphosite-specific antibodies featured on the microarray. Among all 182 tyrosine phosphorylated target proteins found in our assay (Supplementary Table S1), we focused on the proteins that are critically relevant to AD pathogenesis (Figure 5B). Among these proteins, ROCK1 and ROCK2 are known as cytoskeleton and actin dynamics regulators, which have an impact on dendritic spine plasticity and morphology that regulate learning and memory cognitive behaviors [25]. It was previously shown that the expression of both ROCK1 and ROCK2 is increased in AD, and that their inhibition reduces Aβ levels by promoting APP degradation [26,27,28]. Interestingly, both ROCK1 and ROCK2 were found as targets of FAK in our assay (+53% CFC and +192% CFC, respectively). Additionally, abl1 is a non-receptor tyrosine kinase that participates in the regulation of cytoskeletal signaling pathways and was recently found as a direct interactor of Tau. Moreover, increased levels of abl1 in human AD brain samples indicate that it may have an important role in the disease [29]. Importantly, abl1 was found as a potential target of FAK in our assay (Y226, +17% CFC; Y257, +101% CFC; Y264, +6% CFC; Y413, +60% CFC; Y469, +69% CFC). An additional FAK target that was found in our screen is MST1R, a receptor to protein kinase that is crucial in the induction of oxidative stress, which leads to neuronal cell death [30] (Y1238, +114% CFC; Y1238 +1239, +80% CFC).

Tyrosine phosphorylation of GRIN2A (NMDAR2A) and GRIN2B (NMDAR2B), subunits of the NMDA receptor, was significantly increased in our assay (Y943, +77% CFC). Importantly, increased expression and activation of this subunit in AD enhances the pathology of the disorder in mouse models [31]. The insulin-like growth factor-1 receptor (IGF1R) was significantly tyrosine phosphorylated in our assay (Y1346, +54% CFC). IGF1R is an aging regulator known to be associated with AD progression. Moreover, its inhibition mitigates disease phenotype [32]. Both ERBB2 and ERBB3 encode a protein-tyrosine kinase receptor pair that recognizes neuregulin-1 as a ligand, and their alteration is associated with AD phenotypes [33]. Additionally, ERBB2 is known to regulate autophagy and to block the clearing of APP that produces Aβ in AD [34]. Interestingly, we found a significant increase in the phosphorylation of both ERBB2 and ERBB3 in our assay (ERBB2: Y735, +36% CFC; Y877, +66% CFC; Y1248, +43% CFC; ERBB3: Y1289, +98% CFC; Y1307, +39% CFC; Y1328, +65% CFC). TRKB was another receptor-tyrosine kinase that was increased in phosphorylation by FAK (Y702, +532% CFC). Phosphorylation of JAK1 (+61% CFC), JAK2 (Y570, +100% CFC; Y1007+1008, +37% CFC), STAT1 (+57% CFC), and STAT5 (Y694, +305% CFC), components of the JAK-STAT pathway, was significantly increased in hippocampal lysates following the addition of purified active FAK in our assay. Indeed, it was recently shown that inactivation of the JAK-STAT pathway by Aβ leads to memory impairments in AD [35]. Another protein that has a significant role in AD and was found in our assay was cyclin-dependent kinase 5 (CDK5) (+58% CFC). Interestingly, the APP protein is known to be a CDK5 substrate, and increased levels of CDK5 lead to elevation of APP processing and elevated Aβ plaque production while inhibition of CDK5 improves AD symptoms [36].

All the above tyrosine phosphorylated FAK targets indicate that the kinase may have a role in regulating the physiology and mechanisms of AD. To elucidate the pathways and processes which are affected by FAK activity, we performed enrichment analysis using all protein targets that were found in our in-lysate kinase assay (Figure 5C and Supplementary Table S2). Among the pathways that were significantly changed (*p*-value ≤ 0.05, log *p*-value ≥ 1.3) we found several neuronal pathways including glutamatergic synaptic transmission, neuron projection development, dendrite morphogenesis, and axon guidance (Figure 5D). Several cytoskeletal pathways were also significantly enriched in our analysis, including integrin signaling, actin cytoskeleton organization, Rho GTPase activation, regulation of cell adhesion and cell shape, and endocytosis (Figure 5E). As a responder to extracellular stimuli, FAK is known to regulate actin cytoskeleton organization, microtubule dynamics, and cell adhesion by controlling integrin-mediated Rho GTPase activation and signaling [37,38,39]. Therefore, some of the cytoskeletal pathologies observed in our 3xTg-AD mice may be mediated by the hippocampal overexpression of FAK.

Our in-lysate kinase assay analysis revealed additional pathways which are related to AD, such as STAT3-mediated signaling, TNF-mediated signaling, and PI3K signaling (Figure 5E). Importantly, STAT3-mediated signaling is known to play a role in memory, which implicates its involvement in AD [35]. TNF-mediated signaling is a part of the larger insulin signaling pathway in AD. Specifically, TNF is increased in AD due to the accumulation of Aβ, which activates a pathway leading to inhibitory phosphorylation of IRS-1 and reduced phosphorylation in the PI3K-mediated signaling pathway. Reduced PI3K phosphorylation in AD leads to impairments in the insulin signaling pathway and consequent insulin resistance, which correlates with synaptic dysfunctions and impaired memory in AD [40]. Considered together, our in-lysate target and pathway support a significant role for FAK in controlling the AD-like phenotype observed in our 3xTg-AD mouse model by controlling target proteins, which are associated with neuronal, cytoskeletal, PI3K-mediated, TNF-mediated, and STAT3-mediated signal transduction.

### 2.8. Overexpression of FAK Mediates AD-like Phenotypes in 3xTg-AD Mice by Controlling the PI3K and Insulin Signaling Pathways, Re-Entry into the Cell Cycle, and Neuronal Cell Death

**Figure 6:**
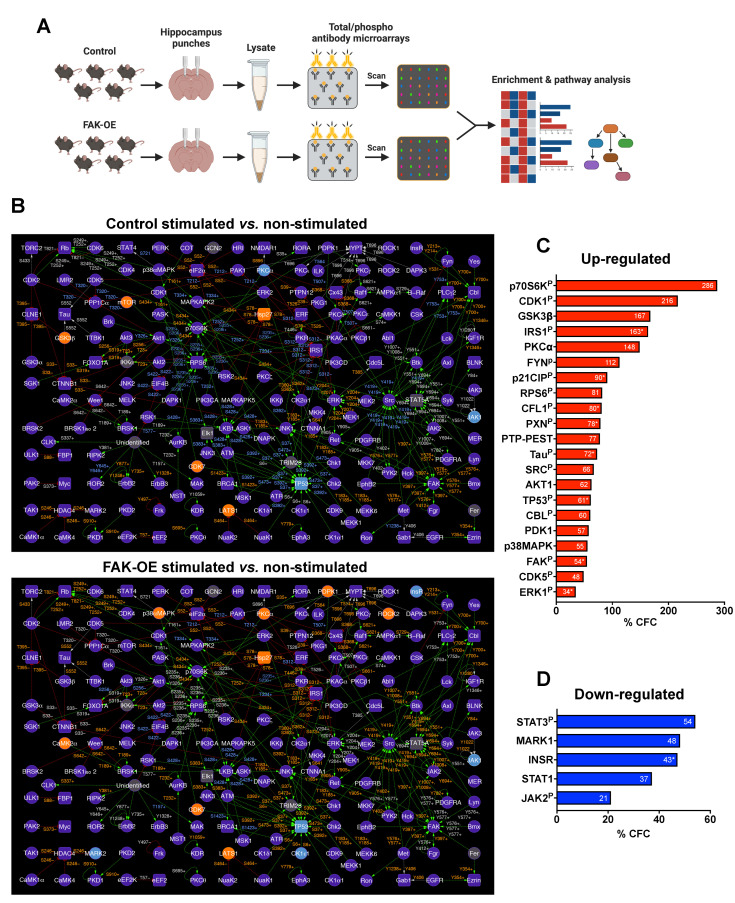
Interaction network maps of hippocampal proteins from control and FAK-OE mice following memory and learning stimulation. (**A**) Diagram of the antibody microarray assay. (**B**) Interaction network maps of hippocampus proteins from control (top map) and FAK-OE (bottom map) mice following learning and memory paradigms, normalized to corresponding non-stimulated mice. Protein nodes which appear in purple represent proteins which had no change in expression. Orange nodes represent proteins with expression increased in 45% CFC or greater. Blue nodes represent proteins with expression decreased in 45% CFC or lower. Grey nodes represent proteins in which expression was not tracked with antibodies on the microarrays. Green lines (edges) represent interactions where a protein kinase phosphorylates a target substrate protein and activates it. Red lines represent interactions where a protein kinase phosphorylates a target substrate protein and inactivates it. Grey lines represent interactions where the effects of protein kinase mediated phosphorylation on the target protein are unknown. Orange color of phosphosite text near connecting edges represent an increase in the phosphorylation of the substrate protein by 45% CFC or greater following stimulation. Blue color of phosphosite text near connecting edges represent a decrease in phosphorylation of a target substrate by 45% CFC or lower following stimulation. Changes in phosphorylation that were less than 45% CFC are depicted in grey text. Protein kinases and protein phosphatases appear as circular nodes. Non-kinase and non-phosphatase proteins appear as square shaped nodes. Kinase-substrate interaction connections are based on in vitro experimental data from the scientific literature, which are documented in the PhosphoNET (www.phosphonet.ca, accessed on 11 July 2022) and KinaseNET (www.kinasenet.ca, accessed on 11 July 2022) databases. (**C**) % CFC values of selected upregulated proteins discussed in the text (FAK-OE stimulated vs. non-stimulated comparison). “P” represents phosphorylated proteins. For proteins with more than one value for the same site, an average % CFC was calculated, and is marked with *. (**D**) % CFC values of selected downregulated proteins discussed in the text (FAK-OE stimulated vs. non-stimulated comparison).

To elucidate the in vivo molecular changes that occur in our 3xTg-AD mice, we performed an antibody microarray assay on hippocampal lysates of control and FAK-OE mice following memory and learning paradigms (Figure 6A). Changes in expression and phosphorylation of proteins in each group were normalized to non-stimulated mice expressing control viral vector or FAK-OE vector. To uncover the biological processes at the system level and reveal possible interactions between protein leads, we built phosphorylation interaction network maps of each of the two groups (Figure 6B). 

Of all the proteins found in our analysis, we selected the proteins that are significantly changed in FAK-OE mice following stimulation, are most relevant to AD pathogenesis, and can explain the AD-like phenotypes observed in this work (Figure 6C,D and Supplementary Table S3). Expression and phosphorylation levels of proteins in the insulin pathway were significantly changed in our pathway. Specifically, we noticed a reduction in insulin receptor (INSR) expression levels in FAK-OE hippocampi compared with control (−47% CFC), which may be attributed to inhibition of the PI3K pathway and/or to the increase in Aβ plaque production in these mice as validated by our amyloid plaque staining shown in Figure 3. Importantly, increased Aβ plaque accumulation in primary hippocampal neurons and in brains of a mouse model of AD is known to trigger aberrant activation of the TNFα/JNK pathway, which results in insulin receptor substrate (IRS1) inhibition [40]. Increased FAK-mediated ERK signaling is also known as leading to impairments in insulin signaling [41,42,43]. In agreement with this, we observed a significant increase in ERK1 phosphorylation (Y204, +64% CFC; T207, +11% CFC; S265, +26% CFC) in hippocampi of FAK-OE mice, which correlates with increased inhibitory serine phosphorylation of IRS1 (S312, +226% CFC; S639, +99% CFC). This inhibitory serine phosphorylation event leads to detachment of IRS1 from insulin receptor and consequently to impairments in insulin-mediated signaling that may also lead to insulin resistance [41,42,43]. 

FAK acts via PI3K-mediated signaling to negatively regulate insulin signaling [44]. Indeed, hippocampal overexpression of FAK led to increased expression of PI3K-phosphoinositide-dependent protein kinase 1 (PDPK1/PDK1) (+57%), which correlated with an increase in AKT1 expression (+62% CFC). Accordingly, a significant increase in GSK3β serine phosphorylation (S9, +167% CFC), which leads to its inactivation, was observed in FAK-OE hippocampi lysates following stimulation. Interestingly, an increase in inhibitory S9 phosphorylation of GSK3β has been previously shown to correlate with elevation of Tau hyperphosphorylation and the formation of neuro-fibrillar tangles in the brain of AD patients [45].

Additionally, p38MAPK is activated by oxidative stress and/or via the PI3K-AKT pathway [46], and has a diverse role in AD pathogenesis, such as mitochondrial and synaptic dysfunction, Tau hyperphosphorylation, apoptosis [47], and re-entry into the cell cycle [48]. Indeed, we found a significant increase in p38MAPK expression in hippocampi lysates of FAK-OE mice (+55% CFC). Moreover, high protein levels of p38MAPK activate TP53, a major regulator of the cell death pathway that takes part in Tau hyperphosphorylation [49] and contributes to neuronal cell death in AD [50]. Our data demonstrated a significant increase in phosphorylation of TP53 (S37, +95% CFC). Aβ leads to FAK-mediated activation of SRC and ERK by inducing un-coordinated cell cycle leading to neuronal death [51]. Indeed, increase in SRC phosphorylation (Y418, +66% CFC) and ERK1 phosphorylation were observed in our assay. Additionally, elevated levels of p21CIP phosphorylation (T145, +105% CFC; S146, +75% CFC) were observed in our screen. Moreover, p21CIP regulates CDC2 (CDK1), which regulates the cell cycle progression, and its high levels may lead to neuronal cell death [52]. Accordingly, high levels of inhibitory CDK1 phosphorylation (Y15, +216% CFC) were observed in our assay.

Over expression of FAK leads to its induced activation (Y397, +87% CFC; Y577, +20% CFC) and phosphorylation of the focal adhesion protein Paxillin (PXN; Y31, +48%; Y118, +107% CFC). Significantly, high phosphorylation of FAK and PXN are observed in dystrophic neurites around plaque cores in AD [14]. Furthermore, association of FYN (Y213+Y214, +112% CFC) and CBL (Y700, +60% CFC) with PXN activates CDK5 (Y15, +49% CFC), which leads to increased APP expression [36]. FYN indirectly promotes the inhibitory serine phosphorylation of GSK3β, which together with CDK5 lead to Tau hyperphosphorylation (S396, +180%; S516, +28%; S713, +38%; S721, +69%; S739, +63%; T522, +52%). PTP-PEST is a protein-tyrosine phosphatase that participates in the focal adhesion signaling cascade and acts as a regulator of the actin cytoskeleton. Indeed, high levels of PTP-PEST negatively regulate cytoskeletal remodeling [53]. Both FAK and PTP-PEST are involved in cytoskeletal signaling which leads to fibrillar Aβ-induced cell death [16]. Our results clearly indicated a significant increase in serine phosphorylation of PTP-PEST (S39, +77%). Another altered cytoskeletal protein that was found in our antibody microarray screen was cofilin (CFL1), which becomes active following de-phosphorylation of its serine-3 residue. A significant increase in phosphorylation of this site was observed in our assay, which may imply that the protein is downregulated in FAK overexpressing mouse hippocampi (S3, +113% CFC). Cofilin is known as a critical protein that regulates AD pathogenesis by forming cofilin-actin rods, which are a hallmark of the disease, mitochondrial dysfunction, excessive actin dynamics that leads to synaptic damage, and microtubule instability that leads to tauopathy [54,55]. Microtubule affinity regulating kinase 1 (MARK1; -48% CFC) acts upstream to and phosphorylates DIXDC1, a protein which regulates cytoskeletal dynamics and controls dendritic morphology. Downregulation of MARK1 following FAK overexpression, as observed in our assay, may reduce DIXDC1 phosphorylation, thereby inhibiting cytoskeletal remodeling [56].

Protein kinase C alpha (PKCα) is necessary for producing synaptic loss by Aβ, which leads to learning and memory deficits by contributing to human late-onset Alzheimer’s disease [57]. Increased levels of both FAK and PKCα may interrupt with dendritic arborization [58]. Indeed, we observed a significant increase in expression levels of this protein in our system (+148% CFC), which may support the observed phenotype in FAK-OE mice.

The 70 kDa S6 kinase (p70S6K; T252, +286% CFC) plays a critical role in regulating cell cycle, cell growth, and cell proliferation [59], and can be activated by the PI3K pathway via PDK1 which showed increased expression in our assay or by Aβ deposition that is enhanced in AD [60]. Interestingly, it was previously shown that p70S6K is associated with increased Tau translation and hyperphosphorylation and accumulation of neurofibrillary tangles. [61]. Additionally, p70S6K can further phosphorylate the ribosomal protein S6 (RPS6) (S236, +81% CFC), and reduction of the levels of p70S6K has been shown to improve the AD-like phenotype of 3xTg-AD mice [62].

While tyrosine phosphorylation of two subunits of the NMDA receptor (NMDAR2A, NMDAR2B) was significantly increased in our kinase assay, a comparison of control mice (stimulated vs. non-stimulated group) to FAK-OE (stimulated vs. non-stimulated group) showed reduced tyrosine phosphorylation in the FAK-OE group (NMDAR2A: Y943, −14% CFC in FAK-OE vs. −3% CFC in control; NMDAR2B: Y1474, +22% CFC vs. +163 in control). This result may imply that although NMDAR subunits appear in vitro as potential targets of FAK, in vivo, the overexpression of FAK indirectly reduces the phosphorylation of NMDAR subunits by activating tyrosine phosphatases. Indeed, it was previously suggested that Aβ accumulation activates protein phosphatase 2B (PP2B) and STEP via the α7 nicotinic receptor. This activation leads to de-phosphorylation of NMDAR subunits and their endocytosis, reduced surface expression, and consequent dysfunction that results in memory loss in AD mice [63]. Our results support a hypothesis by which overexpression of FAK leads to de-phosphorylation-mediated endocytosis of NMDAR subunits, which results in memory and learning impairments of the FAK-OE mice. This hypothesis is further supported by the enrichment of endocytosis pathways in our in-lysate kinase assay (Figure 5E).

Reduced JAK-STAT mediated signaling was observed in our study. Specifically, STAT3 phosphorylation (S727, −54%), STAT1 total expression levels (−37% CFC), and JAK2 phosphorylation (Y570, −21% CFC) were decreased in FAK-OE stimulated mice. Indeed, inactivation of the JAK-STAT pathway by Aβ deposition in AD results in memory impairment [35]. Additionally, this pathway is critical for regulating astrogliosis, and its blocking leads to impaired astrocyte differentiation [64], as was validated in our histological staining of FAK-OE astrocytes.

**Figure 7:**
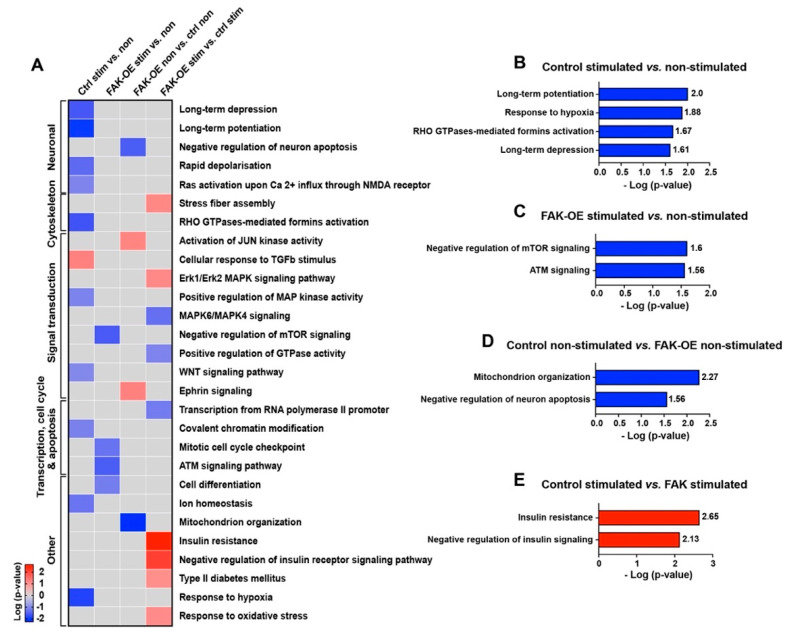
Overexpression of FAK mediates AD-like phenotypes in 3xTg-AD mice by controlling the PI3K and insulin signaling pathways, re-entry into the cell cycle, and neuronal cell death. (**A**) Heatmap of enriched pathways from the antibody microarray assay. Pathways that showed a change in only one group and contained five proteins or more are presented in the heatmap. (**B**–**E**) Selected enriched pathways in each of the four pairwise comparisons: Control stimulated vs. non-stimulated (**B**), FAK-OE stimulated vs. non-stimulated (**C**), FAK-OE non-stimulated vs. control non-stimulated (**D**), and FAK-OE stimulated vs. control stimulated (**E**). Pathways with a score of *p* ≤ 0.5 (log *p*-value ≥ 1.3) were selected and are discussed in the text.

To elucidate the signaling pathways by which overexpression of FAK leads to enhancement of the AD-like phenotype in 3xTg-AD mice, we performed enrichment analysis of the proteins in all four groups (control non-stimulated, control stimulated, FAK-OE non-stimulated, and FAK-OE stimulated) which were pairwise compared. Then, we focused on pathways which are significantly changed (*p*-value ≤ 0.05, log *p*-value ≥ 1.3), unique to one group only, are relevant to AD pathogenesis, and can explain our phenotype (Figure 7A and Supplementary Table S4). In addition to pathways which also appeared in the enrichment analysis of our in-lysate kinase assay, several novel pathways were enriched in the antibody microarray analysis. Among the pathways that significantly changed in the control stimulated vs. non-stimulated comparison (hippocampal pathways which are activated in 3xTg-AD mice during memory and learning), we found downregulation of long-term potentiation, response to hypoxia, Rho GTPase-mediated formins activation, and long-term depression (Figure 7B). In the FAK-OE stimulated vs. non-stimulated comparison (pathways that are changed in FAK overexpressing mice during memory and learning), reduced negative regulation of mTOR signaling and downregulation of ATM signaling were observed (Figure 7C). In the FAK-OE non-stimulated vs. control non-stimulated comparison (pathways which are changed due to the hippocampal overexpression of FAK in resting state), we noticed downregulation of mitochondrion organization and decreased negative regulation of neuronal apoptosis (increased apoptosis of neurons) (Figure 7D). In the FAK-OE stimulated vs. control stimulated comparison (differences between control and FAK-OE hippocampi in processes that are activated during memory and learning stimulation), we observed enrichment of negative regulation of insulin receptor signaling pathway and a significant upregulation of insulin resistance (Figure 7E). Overall, our microarray analysis confirms the involvement of FAK in AD-associated molecular cascades while exposing additional novel FAK-mediated pathways and processes, such as ATM signaling, neuronal apoptosis, and insulin resistance, which may explain the AD-like pathological phenotypes observed in this study.

## 3. Discussion

**Figure 8:**
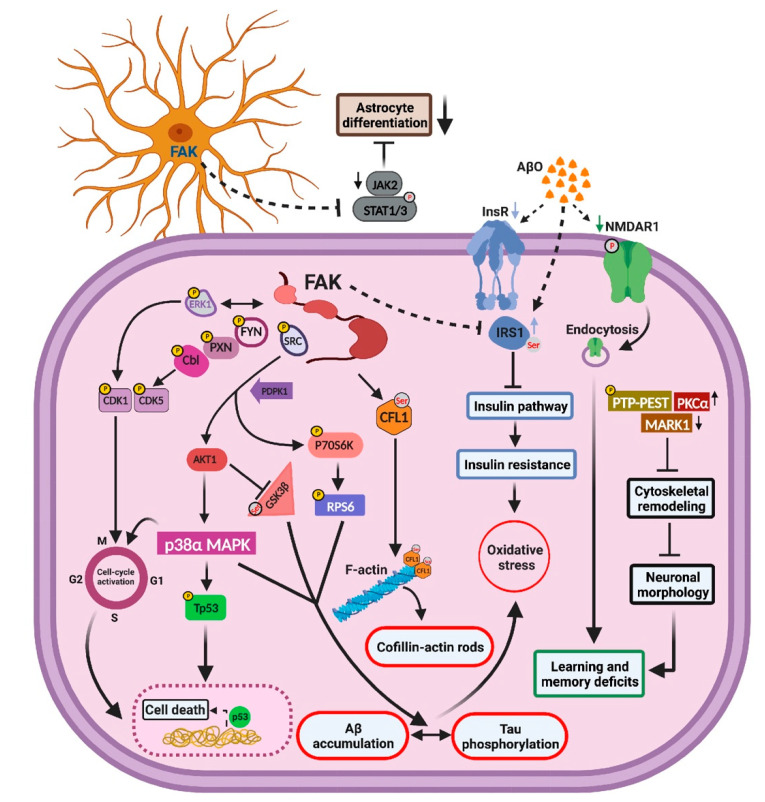
Schematic representation of the hippocampal molecular pathways controlled by FAK in the 3xTg-AD mouse model. FAK regulates hippocampal signaling pathways leading cytoskeletal remodeling that affects neuronal morphology. FAK blocks astrocyte differentiation via the JAK-STAT pathway and increases Tau hyperphosphorylation through PI3K/AKT and RPS6 signaling cascades. FAK activates cell cycle re-entry which leads to neuronal cell death by controlling CDKs and p38α-MAPK, while downregulating the insulin signaling pathway, thereby increasing insulin resistance and leading to oxidative stress. “P” represents tyrosine phosphorylation of a protein; “Ser” represents serine phosphorylation. Arrows represent increase or decrease in expression or phosphorylation of specific proteins, as indicated by our antibody microarrays.

Tyrosine phosphorylation and activation of FAK are observed in AD patient brains and in primary cortical cultures treated with Aβ oligomers, where it cooperates with Fyn and PI3K to activate MAPK, indicating that FAK-mediated signaling may contribute to AD pathology. Despite these and other evidence implicating a role for FAK in AD, the molecular and signaling pathways by which FAK regulates AD pathology have not been extensively defined to date. Here, we demonstrate that FAK mediates AD-like pathology by regulating neuronal and cytoskeletal pathways, by enhancing Tau hyperphosphorylation, and by pushing neurons into re-entry into the cell cycle which is followed by cell death. FAK also negatively regulates insulin signaling leading to insulin resistance and affects astrocyte differentiation by downregulating JAK-STAT-mediated signaling. Analysis of the molecular changes and signaling mechanisms associated with FAK overexpression revealed a multitude of down and upregulated proteins and pathways correlated with AD-like phenotypes. Integration of these molecular and signaling findings led us to suggest a mechanistic model for the role of FAK in hippocampus-mediated functions of AD-like mice (Figure 8).

### 3.1. Overexpression of FAK Leads to Decreased Basal Neurite Arborization

Our results showed a significant decrease in dendritic arborization of basal, but not apical, regions of hippocampal pyramidal neurons following overexpression of FAK, while no differences were observed in spine density or in the types of dendritic spines formed in the basal or apical regions (Figure 3). Indeed, a previous study demonstrated that the basal and apical dendrites of mouse hippocampus pyramidal cells differ significantly in both extracellular and intracellular cascades that regulate synaptic plasticity [22]. In agreement with this observation, stronger LTP is found in basal regions compared with apical regions of CA1 pyramidal cells [65]. We suggest that changes in the cytoskeletal proteins PTP-PEST, PKCα, and MARK1, which were observed in our study (Figure 6), may contribute towards the altered neuronal morphology in FAK overexpressing hippocampi. PTP-PEST is a known regulator of cytoskeletal remodeling and cytoskeleton-associated proteins. Specifically, high activation of PTP-PEST impairs Ephrin-induced cytoskeletal remodeling by affecting focal adhesion proteins [53]. FAK-mediated activation of PKCα leads to hyperphosphorylation of the actin filament cross linking protein MARCKS, which results in reduced dendritic arborization [58]. Decrease in levels of the microtubule dynamics regulator protein-serine/threonine kinase MARK1 blocks cytoskeletal remodeling due to the decrease in the phosphorylation of the cytoskeletal dynamics regulator DIXDC1. This decrease further leads to impairment in dendritic morphology [56]. Considered together, our findings indicate that FAK may regulate specific cytoskeletal signaling pathways in the basal part of hippocampal neurons, and that its overexpression leads to reduced arborization of these neuronal regions.

### 3.2. FAK Is Involved in the Hyperphosphorylation of Tau

Aβ accumulation and Tau hyperphosphorylation are among the major hallmarks of AD. Increase in PI3K-AKT signaling along with increased active form of GSK3β are known to harness the delicate balance between the normal phosphorylation and the pathological hyperphosphorylation of Tau [45]. 

Our data demonstrated the activation of this pathway indicating that FAK indirectly contributes to Tau hyperphosphorylation. RPS6 and the stress-induced kinase p38MAPK, which is activated by the PI3K-AKT pathway, are also known to regulate the balance between phosphorylation and hyperphosphorylation of Tau [47,61], and are both upregulated in our study (Figure 6). Considered together, our data support a model by which overexpression of FAK leads to increased hyperphosphorylation of Tau by several mechanisms including p38MAPK, GSK3β, and RPS6, and consequently to the enhanced AD-like phenotype observed in FAK-OE mice. Interestingly, Tau was also found as a tyrosine phosphorylation target in our in-lysate kinase assay, indicating that it may be a direct substrate of FAK or a FAK-associated tyrosine kinase. To our knowledge, this is the first documentation of the link between FAK and Tau phosphorylation in AD.

### 3.3. FAK-Mediated Downregulation of JAK-STAT Pathway Leads to Reduced Astrogliosis

Astrocytes are important central nervous system resident cells and serve several functions, such as providing structural support for neuronal cells and maintenance of homeostatic balance. Moreover, recent studies indicate that changes in astrocyte structure or function influence neuronal morphology. In AD, the presence of Aβ disrupts glial transmission and neurotransmitter uptake and alters calcium signaling in astrocytes. Furthermore, astrocytes express apolipoprotein E and are involved in the production, degradation, and removal of Aβ. As a result, astrocytes could play a key role in the early stages of the disease and changes in the function of these cells could lead to neurodegeneration. Indeed, it was recently shown that astrocytes in AD patients manifest many pathological changes typical of AD [66,67]. A decrease in both cell density and surface area of astrocytes was observed in our 3xTg-AD mice overexpressing FAK in their hippocampi (Figure 4). Mechanistic investigation of this phenotype revealed alterations in the JAK-STAT pathway (Figure 5 and Figure 6). Importantly, the JAK-STAT pathway plays a critical role in astrogliosis by activation of STAT 1/3 and consequent gene transcription. Accordingly, STAT 1/3 knockout mice show impaired astrocyte differentiation [64]. Considered together, our data indicate that overexpression of FAK in hippocampal astrocytes of 3×Tg-AD mice leads to downregulation of the JAK-STAT pathway in these cells, which contributes to their impaired function and to the AD-like phenotype observed in our study.

### 3.4. FAK Overexpression Regulates Cell Cycle Re-Entry and Consequent Neuronal Cell Death

High metabolic activity of neurons and their intense transcriptional and translational activities generate a large amount of reactive oxygen species with DNA damaging capacity. Healthy neurons ensure their longevity and functionality by several mechanisms, which maintain the integrity of their genomes. One of these mechanisms that exists in healthy individuals is re-entry into the cell cycle followed by activation of DNA repair mechanisms in damaged neurons. AD neurons, which are exposed to high oxidative stress and DNA damage, attempt to re-enter the cell cycle to repair their genomes. However, deficiencies in their DNA repair mechanisms usually lead to neuronal cell death [68]. Indeed, deficiencies in the repair of DNA lesions are observed in neurons from AD patients and may be involved in pathogenesis of the disease [69]. One of the mechanisms that couples cell cycle re-entry to neuronal cell death is ATM signaling. Interestingly, reduced levels and activity of the ATM protein were found in AD patient brains, and a failure of ATM-mediated repair mechanism may be involved in the neuronal death in AD patients [70].

Our data demonstrate increased FAK-mediated phosphorylation and activation of FYN, PXN, CBL, and CDK5 as well as ERK1-mediated activation of CDK1, both are known to activate cell cycle re-entry and neuronal cell death [36,51] (Figure 6). Additionally, we observed a significant increase in phosphorylation of TP53, a known regulator of cell death in general and of neuronal cell death in particular [50]. These data further converge into a significant reduction in ATM-mediated signaling in FAK overexpressing stimulated hippocampi, which is accompanied by an increase in neuronal cell death (Figure 7). Considered together, our data support the hypothesis that following the overexpression of FAK, hippocampal neurons re-enter the cell cycle to repair their accumulated DNA damage, which is already high in 3×Tg-AD mouse brains. However, they fail to do so due to impairments in their DNA repair mechanisms, which leads to TP53-mediated neuronal cell death. These data validate earlier observations that indicated FAK may regulate the activation of G1-S transition and consequent re-entry into the cell cycle, which results in neuronal cell death [52].

### 3.5. Aberrations in Insulin Signaling and Insulin Resistance Are Resulted by FAK Overexpression

Cumulative data over the past decade indicate that the brain is sensitive to insulin as a neurotrophic factor. Indeed, beyond its canonical role in body and brain metabolism regulation, insulin also modifies neuronal activity including synaptic plasticity and learning and memory. Recent studies support impairment of insulin signaling in the brains of AD patients and AD mouse models, and aberrant insulin signaling is linked to AD pathology. Along these lines, decreased amounts of insulin and reduced expression of insulin receptor along with increased serine phosphorylation of IRS1 have been observed in brains from AD patients [71].

In normal brains, insulin binding to its receptor triggers phosphorylation of IRS1, which results in PI3K activation and downstream cellular responses that facilitate neuronal growth and survival, synaptic plasticity, and learning and memory. In AD, accumulation of Aβ oligomers triggers increased activation of TNFα, which activates JNK leading to inhibitory serine phosphorylation of IRS1. Moreover, Aβ oligomers enhance removal of insulin receptors from the cell surface by their internalization. Aberrant insulin signaling and the development of insulin resistance affect the expression of insulin degrading enzyme and consequently Aβ degradation. Furthermore, the reduction in insulin signaling leads to the increase in GSK3β activity, which increases Tau hyperphosphorylation and neurofibrillary tangle production. Along these lines, decreased amounts of insulin and reduced expression of insulin receptor along with increased serine phosphorylation of IRS1 have been observed in brains from AD patients [41].

In agreement with this model, we observed a reduction in insulin receptor expression levels in FAK overexpressing mouse hippocampi, along with increased inhibitory serine phosphorylation of IRS1 and high ERK activity. Our analysis further confirmed the enrichment of TNF-mediated pathway, insulin resistance, and increased ERK signaling along with downregulation of insulin signaling. Considered together, our results provide an in vivo validation to previous findings that FAK acts as a negative regulator of the PI3K-mediated insulin pathway within the brain, and that high neuronal expression of FAK leads to brain insulin resistance. The association of FAK with neuronal insulin resistance highlights its role in mediating AD complications, such as oxidative stress and neuronal death [44], which were also observed in our study.

## 4. Materials and Methods

### 4.1. Animals

Homozygous 3xTg-AD mice carrying three mutations associated with familial Alzheimer’s disease (APP KM670/671NL Swedish, PSEN1 M146V, MAPT P301L) [72] were obtained from Jackson Laboratories (Bar Harbor, ME). The mice are viable, fertile, and display no initial gross physical or behavioral abnormalities, and develop age-related progressive neuropathology including amyloid plaques (which appear by 6 months) and Tau tangles (by 12 to 15 months). Behavioral tests were performed on 8-month-old 3,Tg-AD or non-transgenic C57BL/6 male mice. All mice were bred and maintained in a vivarium at 22 °C in a 12 h light/dark cycle, with food and water available *ad libitum.* Mice were housed according to the Federation of Laboratory Animal Science Associations (FELASA) guidelines, and all the experimental protocols were approved by the Institutional Animal Care and Use Committee (IACUC) at Bar-Ilan University, Faculty of Medicine (protocol number 60-09-2019).

### 4.2. Quantitative Real-Time PCR (qRT-PCR)

Five hippocampus tissue punches were collected from five mice in each group of 1/4/8-month-old 3xTg-AD mice and non-transgenic mice. Total RNA was isolated using the RNeasy Mini Kit (QIAGEN) according to manufacturer’s instructions and including DNase treatment, and RNA concentration was measured using NanoDrop 2000c spectrophotometer (Thermo Fisher Scientific, Waltham, MA, USA). cDNA was prepared from total RNA using high-capacity cDNA reverse transcription kit (Thermo Fisher Scientific) following the manufacturer’s instructions. qRT-PCR was performed using Fast SYBR Green Master Mix with HPRT as a control housekeeping gene (Applied Biosystems, Thermo Fisher Scientific). The reaction was set in triplicates of 20 μL volume using 10 ng of cDNA template. Data were analyzed in StepOnePlus system and installed with StepOne Software v2.3 (Applied Biosystems). Quantification was performed using the comparative Ct (ΔΔCt) method. The primers used for qRT-PCR are presented in Table 1.

### 4.3. Plasmids

Mouse *Ptk2* and *Ptk2b* cDNAs were isolated from mouse embryonic fibroblast cDNA library using PCR with primers containing FLAG tag and sub-cloned into pCSC-SP-PW-CMV-IRES/GFP vector. For shRNA-mediated knockdown, the following sequence was cloned into pLL3.7 lentiviral plasmid: 5′-GGGCATCATTCAGAAGATA-3′. Expression and specificity of the plasmids were verified by transfection into HEK293T cells. 

### 4.4. Viral Vector Preparation

HEK293T cells were cultured in DMEM/10% FBS. Recombinant lentiviruses were produced by co-transfecting cells with lentiviral plasmids containing *Ptk2* or shRNA sequence along with pDML, pRSV-VSVG, and pCMV-VSVG packaging plasmids using the PEI transfection method. At 24 and 48 h following transfection, cell supernatants were collected and concentrated using ultracentrifugation (20,000 rpm, 15 °C, 150 min). Virus pellets were resuspended in Hanks’ Balanced Salt Solution, aliquoted, and frozen at 80 °C until use. 

### 4.5. Viral Vector Delivery

Viral vectors were delivered into the hippocampi of 8-month-old mice by stereotaxic injection using a Hamilton syringe connected to a motorized nanoinjector. Injections were performed using the following coordinates, relative to Bregma: AP = −1.9 mm, ML = ±1.25 mm, DV = −2.0 mm, based on a calibration study indicating these coordinates as leading to the hippocampus in the C57BL/6 strain on our system. During the surgery period, animals remained on a heating pad and were brought back to their home cages post-surgery while monitoring for 24 h. Mice were allowed to recover for 3 weeks before behavioral testing. To validate the accuracy of injection, hippocampi were immunostained using the procedure as mentioned below.

### 4.6. Immunoblotting

Brain tissue was homogenized in an equal ratio of tissue homogenate buffer (50 mM Tris-HCl pH 7.5, 150 mM KCl, 320 mM sucrose, protease inhibitor cocktail (Sigma-Aldrich, St. Louis, MO, USA) and lysis buffer (1% Triton, 10% glycerol, 120 mM NaCl, 25 mM HEPES, 1 mM EDTA, 0.75 mM MgCl_2_, 2 mM NaF, 1 mM sodium vanadate, protease inhibitor cocktail). Alternatively, transfected HEK293T were washed in ice-cold PBS and lysed in Triton lysis buffer (1% Triton, 10% glycerol, 120 mM NaCl, 25 mM HEPES, 1 mM EDTA, 0.75 mM MgCl_2_, 2 mM NaF, 1 mM sodium orthovanadate, and protease inhibitors). Samples were incubated on ice for 10 min and then centrifuged at 11,000× *g* for 10 min at 4 °C to separate nuclei from residual tissue. Total protein concentration was determined using a DC protein assay (Bio-Rad Laboratories, Hercules, CA, USA), and equal amounts were loaded on SDS-PAGE, transferred to a nitrocellulose membrane, and blocked in an Odyssey blocking buffer. Membranes were incubated with primary and secondary antibodies and imaged using the Odyssey CLx imaging system (LI-COR Biosciences, Lincoln, NE, USA). Anti-FAK (clone 77; 610088) was obtained from BD Biosciences. Anti-FLAG (clone M2; F3165) and anti-β actin (clone AC-15; A5441) were obtained from Sigma-Aldrich. Secondary antibodies (goat anti mouse 680LT and goat anti rabbit 800CW) were obtained from Li-COR Biosciences.

### 4.7. Contextual and Cued Fear Conditioning

One day prior to memory training, each mouse was habituated to the fear conditioning cage for a period of 5 min. On the training day, each mouse was placed into the conditioning chamber (10.5 × 10.5 × 10.5 cm) and allowed to explore it freely for 2 min. A tone (75 dB) was sounded as the conditional stimulus for 30 s followed by a 2 s mild foot shock (0.7 mA) as the un-conditioned stimulus. Following a 1 min break, another tone followed by a mild foot shock was administered. Mice were returned to the home cage 1 min after the second tone-shock session. At 24 h following the training session, mice were placed back into the conditioning chamber for 5 min and their freezing behavior was measured during this time as a measure of contextual memory. At least 3 h following context testing, mice were placed into a different white Plexiglass chamber with different odor, flooring, and light for cue-dependent memory testing. Following a 2 min habituation, the tone was turned on for 30 s. This was followed by two more periods of 1 min break and a 30 s tone. Freezing during the three tone periods was recorded. 

### 4.8. Morris Water Maze

The Morris water maze paradigm was performed in a circular tank of 130 cm diameter and a hidden platform with 15 cm diameter. The tank was filled with water at 22 ± 2 °C until the platform was submerged to a depth of 1 cm and made opaque with skimmed milk powder. A single mouse was gently placed in one of the four starting locations facing the pool wall and allowed to search the platform for 60 s. Two trials per day were conducted for 5 d with the platform placed in the same quadrant. Latency to reach the platform was recorded during the 5 d of experiment. On day 6, the probe test was conducted by removing the platform, and the mouse was allowed to swim for 60 s to locate the platform. The percentage of time spent in each quadrant and the number of platform crossings were recorded.

### 4.9. Video Tracking Analysis

All behavioral experiments were recorded with a Panasonic WV-CL930 camera and with the Ganz IR 50/50 infrared panel. Analysis of videos was performed using EthoVision XT-10 (Noldus).

### 4.10. Brain Tissue Preparation and Immunofluorescence

Mice were anesthetized intraperitonially (Pentobarbital 30 mg/kg) and perfused intracardially with 4% paraformaldehyde solution pH 9.8. Brains were removed and fixed overnight in fresh paraformaldehyde, and then incubated in 30% sucrose prepared in 4% paraformaldehyde. Brains were sliced in a sliding microtome to produce 30-micron floating sections. For immunofluorescence, brain sections were blocked for 1 h in blocking solution (10% horse serum, 0.3% Triton, and 1XPBS), and then incubated with anti-GFP (Roche) overnight at 4 °C. Next, slices were washed with 1XPBS and incubated for 1 h with secondary antibodies (Alexa 488) at room temperature. Sections were stained for 5 min with Hoechst (Sigma-Aldrich), and washed three times, followed by mounting. Images were taken using Zeiss LSM710 confocal microscope and the images were processed using ImageJ V1.53 software.

### 4.11. Immunohistochemistry

Mice were transcardially perfused with PBS followed by 4% paraformaldehyde. The brains were carefully removed and fixed in 4% paraformaldehyde for 24 h, then placed in 70% ethanol at 4 °C for 48 h. Paraffin-embedded blocks were sliced on a Leica microtome (Leica Biosystems) at a thickness of 4 µm. Brain sections were mounted on slides and air-dried overnight at room temperature. Histochemical analysis was performed on microglia (anti-Iba1, Novus Biologicals, #NB100-1028), astrocytes (anti-GFAP, Biolegend, #835301). Five brain coronal sections per mouse were stained. Five serial sections at a thickness of 4 μm were cut at 20 μm intervals throughout the brain and were labeled with anti-Iba1 and anti-GFAP. A Leica Refine-HRP kit (Leica Biosystems Newcastle Ltd., UK) was used for detection and counterstaining with hematoxylin. The sections were mounted and viewed under an Axio Scan.Z1 (Zeiss, Oberkochen, Germany) fluorescent and bright-field slide scanner with a 40X/0.95 NA objective. Images were acquired with a Z-stack of 0.5 μm. Additionally, an Axio Imager 2 Upright ApoTome microscope was used to capture images with a 100X/1.4 NA oil immersion objective. Immunolabeling was performed in the corresponding hippocampal areas, and image analysis was carried out using Zen Blue 2.5 software (Zeiss, Oberkochen, Germany) with fixed background intensity threshold for all sections representing a single type of staining.

### 4.12. Golgi-Cox Staining

Brains of *Pyk2*-KD and control mice were dissected and immediately stained using the instructions mentioned in the manual of the Super Golgi Kit (Bioeeno Tech, Santa Ana, CA, USA). After the initial Golgi staining steps, the brains were sectioned into slices of 150 μm thick using a Leica CM 1900 Cryostat. Furthermore, these sections were stained according to the manufacturer’s instructions and slides were mounted using Ecomount from Kaltek (Cat #0605). Stack images of whole-mount sections of cortex, CA1, and DG hippocampal region were acquired using the bright field ApoTome microscope from Zeiss. Dendritic arbor length, Sholl analysis, and spine number were analyzed using Imaris 8.1 software (Bitplane AG, Zurich, Switzerland) as described previously [73].

### 4.13. Antibody Microarrays 

Hippocampal punches were collected from control and *Ptk2*-OE injected mice at the end of behavioral tests and subjected to Kinexus Kinex^TM^ KAM-1325 antibody microarray analyses as described [74]. These microarrays feature about 1350 commercial antibodies produced principally by Kinexus as well as from other suppliers following their in-house validation, with each antibody printed in quadruplicate on each Nexterion P slide (Schott AG, Jena, Germany). Briefly, lysates from each sample were incubated at pH 9.0 with 400 mM Tris (2-carboxyethyl) phosphaine hydrochloride (TCEP) to reduce disulfide linkages for 30 min at 37 °C and then 6 mM 2-nitro-5-thiocyanatobenzoic acid (NTCB) to cleave proteins after cysteine residues for 30 min at 37 °C. Chemically cleaved lysate proteins (100 μg) were subsequently covalently labeled with Sulfo-NHS-biotin (50 µg) (A8001, ApexBio, Houston, TX, USA) for 1 h. Free biotin molecules were removed via gel filtration. After blocking nonspecific binding sites on the array, an incubation chamber was mounted onto the microarray to permit the loading of the biotinylated, cleaved protein samples. After incubation for 2 h at 20 °C, unbound proteins were washed. Then, the microarray was incubated for 12 min at 20 °C with anti-biotin goat polyclonal antibody (10 µg) (B3640-1MG, Millipore-Sigma, St. Louis, MO, USA) that was previously labeled with a 50/50 dye mixture of Alexa Fluor 546 dye (A20002, Thermo Fisher Scientific, Rockford, IL, USA) and Sulfo-Cyanine3 dye (11320, Lumiprobe, Hannover, Germany) for 1 h. Thereafter, two 16-bit images from each array were captured using a ScanArray Reader (Perkin-Elmer). An antibody array was performed in parallel on four different chips. The output of the array consisted of the average normalized net signals (i.e., the average of four normalized net signal values of each antibody on the microarray). Standard deviation and percent standard deviation of four separate measurements of globally normalized signal intensity values for each different antibody on the microarray were calculated. Data are presented as percent change from control (% CFC). A positive value corresponds to an increase in signal intensity in response to the treatment, with a value of 100% corresponding to a 2-fold increment in signal intensity. A negative CFC value indicates the degree of reduction in signal intensity from the control. Each parameter has its Student’s *t*-test *p*-value, which is the probability (*p*) value that there is no difference between the control and test samples. A *p*-value was determined with n = 4 measurements in each set, which were paired and 2-tailed in distribution. 

### 4.14. In-Lysate Kinase Assay

Lysates from five individual brain hippocampi of 3xTg-AD control mice were pooled. Pooled lysate proteins were initially chemically cleaved at cysteine residues with TCEP and NTCB at pH 9, and then buffer-exchanged following passage through a 0.5 mL Sephadex G25 spin column. Next, the preparation was incubated in a 37 °C water bath for 30 min with a buffer that contained 500 μM ATP with or without 0.5 μg of preparations of active, human recombinant FAK. At the end of incubation, reactive biotin was added, and the peptides were biotinylated. Excess biotin was removed with a 0.5 mL Sephadex G25 spin column, and approximately 25 μg of total lysate were separately incubated with KAM-1325 antibody microarrays. Detection of captured lysate peptides on the microarrays was achieved with fluorescent dye-labeled anti-biotin reporter antibodies and scanning with a ScanArray instrument (Perkin-Elmer).

### 4.15. Enrichment Analysis

Total and phosphorylated proteins with a *p*-value of ≤ 0.05 from KAM-1325 protein microarrays were selected for enrichment analysis using DAVID 6.8. All proteins contained within the KAM-1325 microarray were used as background. 

### 4.16. Statistical Analysis

Statistical analysis was performed using GraphPad Prism 8.0. Statistical significance was calculated using Student’s *t*-test or two-way ANOVA with the Bonferroni post hoc test as appropriate. In all graphs, error bars represent standard errors of the mean (SEM).

## 5. Conclusions

In conclusion, despite accumulating data that implicate a role for FAK in AD, the specific mechanisms and pathways by which this kinase acts were largely unknown. Using the 3XTg-AD mouse model combined with high-throughput proteomics, we showed here, for the first time, that overexpression of FAK leads to cognitive impairments accompanied by Aβ overload, altered neuronal morphology, and deficient astrocyte differentiation. Overall, our results validate previous findings that point to a role for FAK in AD and add significant knowledge regarding the mechanisms and pathways by which FAK regulates AD pathology. Understanding the molecular mechanisms and signaling pathways by which FAK is involved in AD may lead to the development of novel strategies for slowing or stopping the disease.

## Figures and Tables

**Table 1:** Primers used for qRT-PCR.

Primer	Sequence
FAK Fwd	5′-CGT GAA GCC TTT TCA AGG AG-3′
FAK Rev	5′-GCA CCT TCT CCT CCT CCA G-3′
HPRT Fwd	5′-GCA GTA CAG CCC CAA AAT GG-3′
HPRT Rev	5′-GGT CCT TTT CAC CAG CAA GCT-3′

## Data Availability

Not applicable.

## Supplementary Materials

The following supporting information can be downloaded at https://www.mdpi.com/article/10.3390/ijms23169055/s1.
